# Divergent eye–brain–heart fatigue signatures during sustained simulated agricultural operations

**DOI:** 10.3389/fphys.2026.1871218

**Published:** 2026-07-08

**Authors:** Zhizheng Hu, Xiangyu Liu, Zhende Jiang, Chun Wang, Zhihui Qian, Lei Ren, Hailin Kui

**Affiliations:** 1 College of Biological and Agricultural Engineering, Jilin University, Changchun, Jilin, China; 2 Orthopaedic Medical Center, The Second Hospital of Jilin University, Changchun, Jilin, China; 3 Key Laboratory of Bionics Engineering, Ministry of Education, Jilin University, Changchun, Jilin, China

**Keywords:** adaptive interfaces, agricultural ergonomics, cognitive fatigue, electroencephalography, eye-tracking, human-machine interaction, multimodal physiological measurement, neuroergonomics

## Abstract

Decoding the dynamic physiological responses of the human central and autonomic nervous systems to sustained cognitive loads is essential for occupational health and neuroergonomics. This study investigates the multidimensional neurophysiological mechanisms of fatigue during complex human-machine interactions, utilizing a simulated agricultural operation paradigm. We developed a multimodal “eye-brain-heart” physio-logging framework, synchronously recording 21-channel EEG, ECG, and pupillometry in 12 experienced subjects over a continuous 5-hour protocol. Given the controlled small-sample and male-only cohort, the present study was positioned as an exploratory within-subject investigation rather than a definitive validation study. Cross-channel coupling analysis suggested task-associated divergence in multimodal physiological fatigue signatures, which was not fully consistent with a simple monolithic accumulation pattern. High-demand interactive tasks induced an “active overload” state, associated with sustained prefrontal beta synchronization, continuous pupillary dilation consistent with elevated cognitive arousal, and sympathetic dominance. Conversely, monotonous monitoring tasks triggered “passive deprivation, “ marked by physiological patterns consistent with task disengagement, prominent parieto-occipital alpha bursts, and a transition to parasympathetic vagal control. These findings provide preliminary evidence that, under the present experimental conditions, occupational cognitive fatigue may be better described as a context-specific neurophysiological divergence rather than a uniform vigilance decrement. The proposed multimodal fingerprint may help reduce the diagnostic ambiguity of single-channel assessments, providing an exploratory neuroergonomic basis for adaptive interventions to preserve optimal physiological states in high-demand operational environments. Further validation in larger, gender-diverse, and more heterogeneous operator cohorts is required before generalizing these findings to broader agricultural populations.

## 1 Introduction

The modern agricultural sector is increasingly characterized by the deployment of intelligent and complex machinery. While physical exertion has been substantially alleviated by advanced automation, the cognitive demands placed on operators have increased unprecedentedly (Elliott et al., 2022). During field operations, a severe cognitive load is imposed as operators are required to continuously monitor dynamic multi-screen interfaces, precisely coordinate implements, and adapt to unpredictable terrain (Hwang and Nam, 2023). Particularly during the busy farming season, the combination of extreme weather conditions, hazard-prone working environments, and high-intensity repetitive operations exacerbates operator stress, rapidly accelerating the onset of mental fatigue (Lu et al., 2020; Liu et al., 2022). Prolonged exposure to these high-demand human-machine interaction environments inevitably induces acute operator fatigue. Within agricultural settings, such fatigue is not merely an occupational discomfort; rather, it is recognized as a critical vulnerability that severely compromises operational precision, decision-making capacity, and overall efficiency, thereby significantly elevating the risk of severe safety incidents (Gao et al., 2021). Consequently, the objective quantification and mitigation of operator fatigue have been established as paramount priorities in contemporary neuroergonomic and occupational safety research.

Historically, operator fatigue assessment has predominantly hinged on subjective questionnaires and single-modality physiological monitoring. While self-reporting instruments, such as the NASA-TLX, yield valuable psychological baselines, they are intrinsically constrained by retrospective recall bias and an inability to capture transient, real-time physiological fluctuations during task execution (Tao et al., 2019; Tierney et al., 2025). Consequently, objective monitoring utilizing isolated physiological signals—most notably EEG or ECG—has been extensively adopted. Conventional single-modality models generally correlate elevated low-frequency EEG power with progressive drowsiness (Hu et al., 2024), or rely on HRV metrics to index autonomic shifts (Charles and Nixon, 2019; Lu et al., 2022). However, these traditional methodologies exhibit critical limitations when applied to the highly complex environments of modern agriculture. Crucially, they overlook the profound modulatory effects of distinct task properties, operating under the simplified assumption of a unidirectional, time-dependent fatigue accumulation (Behrens et al., 2023). Furthermore, isolated metrics inevitably suffer from “physiological ambiguity”; for instance, uncalibrated HRV indices or EEG spectral shifts cannot reliably differentiate between physical exertion and cognitive overload (Imran et al., 2024). This ambiguity impedes the precise delineation of task-specific fatigue mechanisms, necessitating a more integrative analytical paradigm.

To circumvent the intrinsic limitations of single-modality assessments, multimodal physiological integration has emerged as a robust paradigm to comprehensively decode human cognitive states (Lohani et al., 2019). Specifically, the integration of ocular, cerebral, and autonomic nervous system metrics—conceptualized as the “Eye-Brain-Heart” triad—provides a multidimensional physiological fingerprint capable of resolving the ambiguity inherent in isolated signals (Giannakakis et al., 2022; Peng et al., 2024). Despite these methodological advancements in continuous monitoring, the underlying neurophysiological mechanisms governing operator fatigue in distinct agricultural tasks remain insufficiently elucidated. Current literature predominantly models agricultural driving as a homogeneous source of cognitive depletion. However, field operations are fundamentally heterogeneous, encompassing tasks that range from highly interactive, continuous control (e.g., implement alignment during baling) to constrained, monotonous monitoring (e.g., rotary tilling) (Saxby et al., 2013; Hu et al., 2026). Crucially, there is a profound lack of deep exploration into how differing task attributes trigger divergent neural pathways. Drawing upon neurocognitive theories of attention, it is hypothesized that highly demanding tasks induce an “active overload” state associated with elevated cognitive arousal and sympathetic modulation, which may be related to LC-NE arousal processes, whereas monotonous tasks precipitate “passive deprivation” consistent with reduced external-task engagement and DMN-related task-unrelated processing tendencies (Hopstaken et al., 2015; Wang et al., 2020). Unveiling this dual-pathway mechanism is critical for accurately mapping task-specific neural responses, yet it remains critically underexplored within the domain of neuroergonomics in agriculture (Matthews and Desmond, 2002).

To bridge these highlighted theoretical and methodological gaps, this study proposes a task-attribute-driven dual-pathway fatigue framework for agricultural operations. A continuous 5-hour simulated agricultural driving experiment, encompassing 12 subjects, was conducted to incorporate three distinct operational paradigms: baling, rotary tilling, and sowing. By simultaneously capturing ocular (pupil diameter, blink frequency), cerebral (Engagement Index, Fatigue Index, frontal beta, and parieto-occipital alpha rhythms), and autonomic (LF/HF ratio and RMSSD) parameters, comprehensive “Eye-Brain-Heart” multimodal physiological fingerprints were constructed. Leveraging these advanced physiological monitoring technologies over an extended continuous duration is intended to explore task-associated temporal changes and cross-channel physiological patterns under sustained agricultural human-machine interaction conditions (Yin and Zhang, 2017; Liu et al., 2023; Zhang et al., 2026). It is examined whether agricultural driver fatigue is not a singular temporal accumulation process, but rather may show a context-dependent divergence: the baling task, requiring high-frequency monitoring and implement alignment, is expected to be associated with an “active overload” pathway characterized by sympathetic dominance (elevated LF/HF ratio, pupil dilation, and increased beta power) (Hu and Min, 2018; Yang et al., 2024). Conversely, the constrained and monotonous nature of the rotary tilling task is expected to be associated with a “passive deprivation” pathway, marked by parasympathetic dominance and EEG/ocular signatures consistent with task disengagement, including alpha band bursts, increased blink frequencies, and elevated RMSSD. By systematically examining these task-associated multimodal signatures, this research aims to provide preliminary neuroergonomic evidence that may inform future validation of context-aware, adaptive human-machine interfaces in next-generation agricultural machinery.

## 2 Materials and methods

### 2.1 Participants

To investigate human-machine interaction during sustained agricultural operations, 12 healthy male participants with prior large tractor driving experience were recruited. All participants were male experienced agricultural machinery operators with relatively homogeneous occupational backgrounds. This controlled cohort was selected to reduce inter-individual variability in baseline physiological responses and task familiarity; however, it also limits the generalizability of the findings to female operators, novice operators, and more heterogeneous agricultural populations. Inclusion criteria required a minimum of 3 years of agricultural operation experience, normal or corrected-to-normal vision without color blindness, and the absence of cardiovascular or neurological disorders.

To ensure baseline physiological consistency, participants were required to maintain sufficient sleep (a minimum of 7 hours) and abstain from alcohol, caffeine, and intense physical activity for 24 hours prior to the experiment. The Pittsburgh Sleep Quality Index (PSQI) was administered to exclude underlying sleep disorders. Prior to the simulated driving tasks, resting heart rate and blood pressure were recorded to establish a stable physiological baseline. The core demographic, anthropometric, and baseline physiological characteristics of the participants are detailed in **Table 1**.

**Table 1:** Demographic and anthropometric characteristics of male participants (N = 12).

Characteristic (Unit)	Mean ± SD	Range
Age (years)	31.4 ± 4.6	24 - 42
Height (cm)	174.5 ± 6.2	165 - 186
Weight (kg)	73.2 ± 8.1	63 - 88
Body Mass Index (BMI)	23.9 ± 1.7	21.2 - 26.5
Tractor driving experience (years)	6.5 ± 2.3	3.5 - 12
Pittsburgh Sleep Quality Index (PSQI) score	3.1 ± 0.8	2 - 4
Resting heart rate (beats/min)	71.5 ± 5.2	64 - 82
Systolic blood pressure (mmHg)	118.4 ± 6.7	105 - 128
Diastolic blood pressure (mmHg)	76.2 ± 5.4	68 - 84

Written informed consent was obtained from all participants after a comprehensive explanation of the study objectives and safety measures. The study was conducted in accordance with the Declaration of Helsinki, and approved by the Ethics Committee of the Second Hospital of Jilin University (protocol code 2025267 on 30 July 2025).

### 2.2 Cyclical task paradigm and apparatus

The experiment was conducted in a light-controlled, acoustic-damped laboratory setting. An ambient illumination of 300 lux and a temperature of 24 ± 1 °C were strictly maintained to eliminate environmental confounders, particularly the pupillary light reflex.

**Figure 1:**
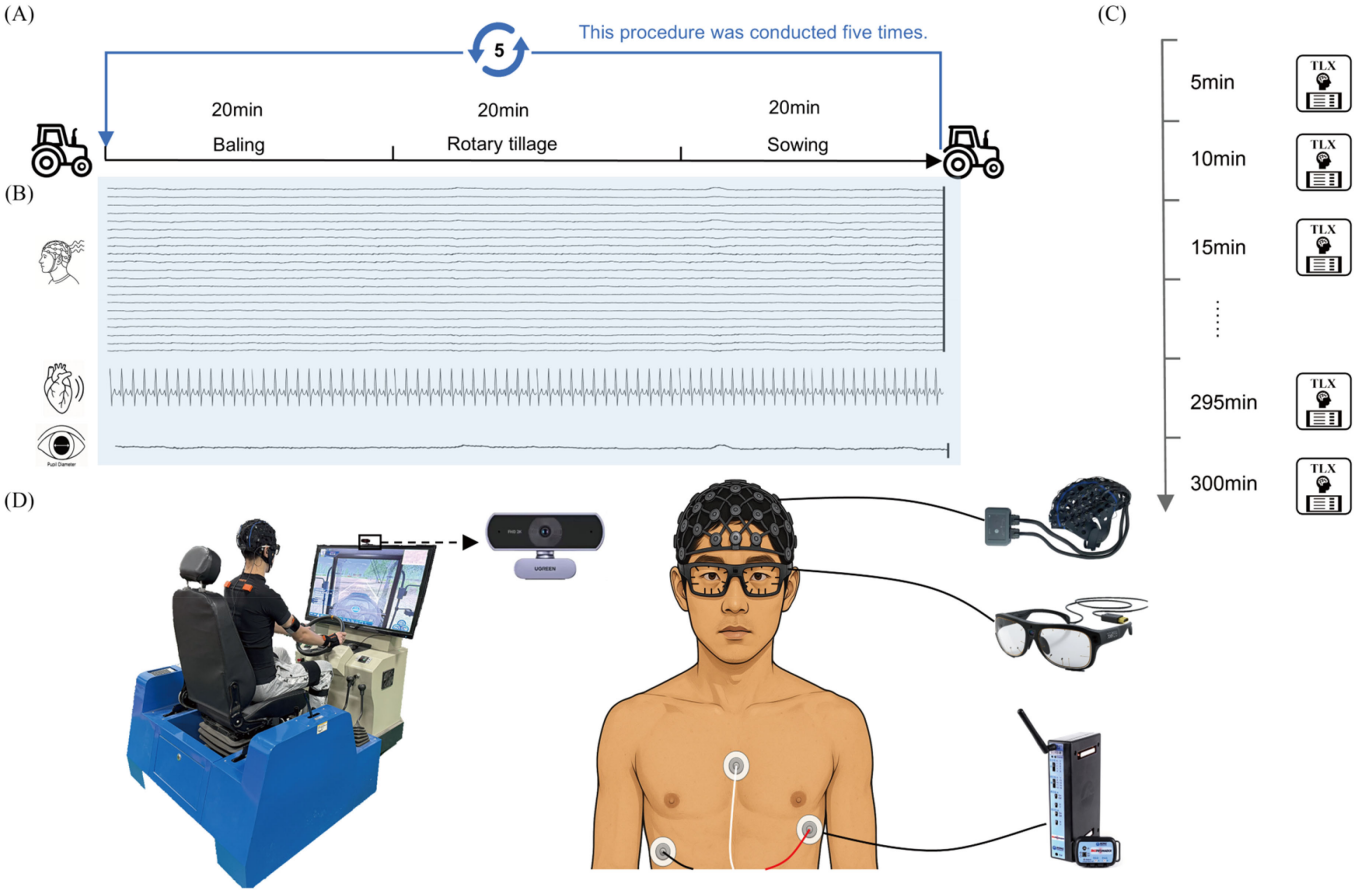
Experimental design and setup. **(A)** The experimental protocol, involving five cycles of three 20-minute agricultural tasks (baling, rotary tillage, and sowing). **(B)** Sample physiological signals recorded, including electroencephalogram (EEG), electrocardiogram (ECG), and pupil diameter. **(C)** Subjective fatigue assessment using the NASA Task Load Index (NASA-TLX). **(D)** The data acquisition setup with a participant in the tractor simulator, illustrating the placement of the key sensors.

As shown in **Figure 1**, participants performed 5 hours of continuous simulated driving to induce varying dimensions of cognitive workload and fatigue. The entire session was divided into 60-minute cycles. Each cycle mapped directly to three sequential 20-minute agricultural tasks: baling, rotary tilling, and sowing. The task sequence was fixed across participants and repeated in the same cyclic order throughout the experiment. This fixed order was adopted to maintain a consistent operational protocol and to approximate a continuous agricultural work sequence in which different task demands occur successively. However, because the order was not randomized or counterbalanced, potential order effects, carryover effects, and time-lapse-related fatigue accumulation cannot be fully excluded. Therefore, the observed differences were interpreted as task-associated physiological patterns under the present fixed cyclic protocol rather than as pure task effects isolated from temporal order.

Multimodal physiological signals were simultaneously acquired using the following high-fidelity apparatus:

- EEG: A wireless SmartBCI headset (Mitsar Co., Ltd., St. Petersburg, Russia; 21 channels, International 10–20 System) with high-input impedance amplifiers (>1 GΩ) to mitigate signal attenuation.
- ECG: A Biopac MP160 system (BIOPAC Systems, Inc., Goleta, CA, USA) utilizing a standard Lead-I configuration.
- Eye-tracking: Wearable Tobii Pro Glasses 3 (Tobii AB, Stockholm, Sweden) for continuous gaze tracking and pupillometry at 100 Hz.

To ensure data integrity during the prolonged recording, the eye-tracker was periodically calibrated, and the EEG DC offset was continuously monitored and maintained within ±50 mV.

### 2.3 Data segmentation and multimodal feature extraction

Continuous physiological streams were preprocessed prior to feature extraction. EEG data were bandpass-filtered (0.5–45 Hz) and subjected to Independent Component Analysis (ICA) to isolate and remove ocular and myogenic artifacts. Bad channels were interpolated, and the data were average re-referenced. ECG signals were processed using a 4th-order Butterworth bandpass filter (0.5–45 Hz) to eliminate baseline wander and high-frequency noise. For eye-tracking data, blink artifacts were removed via linear interpolation and smoothed using a moving average filter.

To preserve the temporal dynamics of cognitive load evolution, the preprocessed data were segmented using a 30-s sliding window with a 5-s step size. Distinct features were extracted across the “eye-brain-heart” modalities to form a comprehensive physiological fingerprint:

- Visual Modality: Average pupil diameter and blink rate.
- Central Nervous System (EEG): Power spectral densities for theta (4–8 Hz), alpha (8–13 Hz), and beta (13–30 Hz) bands were calculated via Fast Fourier Transform. The resulting band-specific spectral powers were used to calculate two EEG-derived spectral indices: the Engagement Index (EI, $P_{\beta }/(P_{\alpha }+P_{\theta })$) and the Fatigue Index (FI, $(P_{\theta }+P_{\alpha })/P_{\beta }$). Here, $P_{\theta }$, $P_{\alpha }$, and $P_{\beta }$denote the spectral power in the theta, alpha, and beta bands, respectively. The theoretical rationale for these indices is that beta-band activity is commonly associated with active cognitive processing and sustained attentional engagement, whereas increased theta/alpha activity is often related to fatigue-related cortical slowing, reduced vigilance, and drowsiness tendency. Therefore, EI and FI were used as operational EEG spectral proxy indicators within the multimodal framework, rather than as direct measurements of latent neurocognitive constructs. To avoid overinterpretation, the EEG-derived EI and FI were interpreted in combination with ocular and autonomic indicators, rather than being used as standalone evidence for a specific neural pathway.
- Autonomic Nervous System (ECG): The Pan-Tompkins algorithm detected R-peaks to compute R-R intervals. Frequency-domain Low-Frequency/High-Frequency (LF/HF) ratio and time-domain Root Mean Square of Successive Differences (RMSSD) were extracted.

The definitions and physical meanings of these multidimensional metrics in the context of human-machine ergonomics are systematically summarized in **Table 2**. Furthermore, following each 20-minute task block, the NASA Task Load Index (NASA-TLX) was administered to capture subjective multidimensional workload dimensions.

**Table 2:** Definitions and ergonomic interpretations of multimodal eye–brain–heart features.

Modality	Feature	Calculation/definition	Ergonomic significance & physical meaning
Eye-tracking	Pupil Diameter	Mean diameter of the pupil (mm) over the task block	Reflects cognitive arousal and mental workload. Dilation typically indicates active information processing or high cognitive demand.
	Blink Rate	Number of blinks per minute (blinks/min)	Associated with visual fatigue and task monotony. A significant increase points to passive task disengagement and drowsiness.
EEG	Engagement Index (EI)	$P_{\beta }/(P_{\alpha }+P_{\theta })$	Used as an EEG spectral proxy for task engagement and sustained attentional allocation. Higher values indicate a relative dominance of beta-band activity over theta/alpha activity, which is commonly associated with active cognitive processing and increased attentional demand.
	Fatigue Index (FI)	$(P_{\alpha }+P_{\theta })/P_{\beta }$	Used as an EEG spectral proxy for fatigue-related cortical slowing and reduced task engagement. Higher values indicate a relative increase in theta/alpha activity compared with beta activity, which is typically associated with decreased vigilance, mental fatigue, or drowsiness-related changes.
ECG (HRV)	LF/HF Ratio	Ratio of low-frequency (0.04-0.15 Hz) to high-frequency (0.15-0.4 Hz) power	Represents sympathovagal balance. An elevated ratio indicates sympathetic dominance, often triggered by acute stress or active tension.
	RMSSD	Root mean square of successive R-R interval differences (ms)	Reflects parasympathetic nervous system activity. An increase in RMSSD correlates with physiological relaxation or passive underload.

$P_{\theta }$, $P_{\alpha }$, and $P_{\beta }$ denote the EEG spectral power in the theta (4–8 Hz), alpha (8–13 Hz), and beta (13–30 Hz) bands, respectively. EI and FI were used as operational EEG spectral indices rather than direct measurements of latent neurocognitive constructs.

### 2.4 Statistical analysis

Statistical analyses were executed using Python (version 3.9) utilizing the scipy.stats, pingouin, and stats models libraries. Prior to inferential modeling, continuous physiological features were subjected to a rigorous data cleansing protocol. Outliers—defined as specific continuous data points exceeding ±3 standard deviations from the intra-subject mean—were identified and replaced using piecewise cubic Hermite interpolating polynomials to preserve signal continuity without distorting local dynamics.

To mitigate the confounding effects of inherent inter-subject baseline variability, especially prevalent in EEG spectral power and ECG-derived HRV metrics, all objective physiological features were within-subject normalized using Z-score standardization across the entire experimental session before group-level aggregation. This within-subject normalization was used to reduce inter-individual baseline variability, but it was not intended to compensate for the limited sample size or to remove constraints on statistical power and generalizability. Because no *a priori* power analysis was conducted before participant recruitment, a *post hoc* sensitivity power analysis was added to clarify the inferential boundary of the present dataset.

To explicitly evaluate the main effects and temporal evolution of different agricultural task properties on the multimodal cognitive load, a Two-way Repeated Measures Analysis of Variance (RM ANOVA) was conducted. The within-subject factors were defined as Task (3 levels: baling, rotary tilling, sowing) and Time Cycle (5 levels: Cycle 1 to Cycle 5). The Shapiro-Wilk test was applied to verify the assumption of normality for the residuals. Mauchly’s test was utilized to assess the assumption of sphericity; in instances of violation (*p* < 0.05), the Greenhouse-Geisser correction was strictly applied to adjust the degrees of freedom. Because the task sequence was fixed rather than randomized or counterbalanced, the Task factor was interpreted as task-associated differences under the present fixed cyclic protocol rather than pure task effects isolated from temporal order. Additional sensitivity analyses, including cycle-wise consistency checks, first-cycle-only analysis, and mixed-effects models including temporal progression, were conducted to evaluate whether the main physiological patterns were preserved after considering potential order and time-lapse effects.

For *post hoc* multiple comparisons exploring significant main effects or interaction effects (Task × Time), Bonferroni corrections were strictly applied to constrain the family-wise Type I error rate. The partial eta-squared ( $\eta_{p}^{2}$) was calculated to report the standardized effect sizes, providing a measure of the magnitude of the observed phenomena independent of sample size. Given the small cohort size, effect sizes were interpreted together with uncertainty estimates rather than as definitive evidence of stable population-level effects. Bootstrap confidence intervals were additionally estimated for key task-related contrasts and effect-size estimates. In addition, linear mixed-effects models with Subject as a random intercept were used as complementary robustness checks for the primary physiological indicators.

Furthermore, to explore the multidimensional coupling mechanisms between subjective psychological perception and objective physiological responses, a Spearman rank-order correlation analysis was conducted across the “eye-brain-heart” feature space and the NASA-TLX subscales. The Benjamini-Hochberg False Discovery Rate (FDR) procedure was applied to adjust the resulting p-values, thereby controlling for false positives in the multiple correlation matrix. The statistical significance threshold for all analyses was pre-defined at *p* < 0.05 *, *p* < 0.01**, *p* < 0.001***.

To explore the organization of the “Eye-Brain-Heart” multimodal feature space and reduce its dimensionality, a Principal Component Analysis (PCA) with Varimax rotation was employed on the aggregated and normalized physiological dataset. This unsupervised multidimensional approach was used as an exploratory dimensionality-reduction procedure to visualize whether the physiological variables tended to group along interpretable axes consistent with the proposed active-overload and passive-deprivation framework. Given the limited sample size, PCA results were interpreted descriptively rather than as confirmatory evidence of latent mechanisms.

## 3 Results

### 3.1 Heterogeneous distribution of subjective multidimensional workload

**Figure 2:**
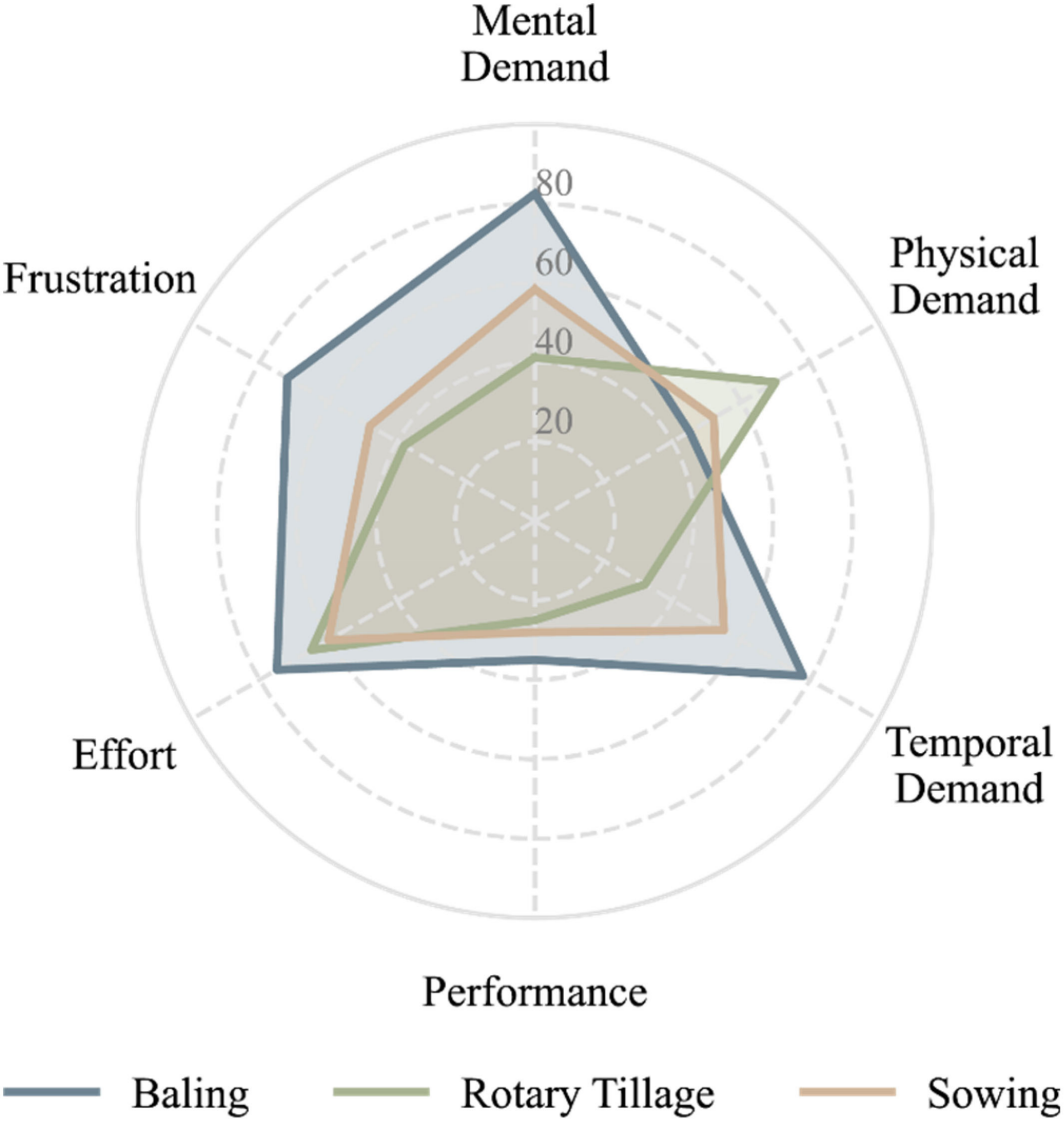
Subjective multidimensional workload scores across six NASA-TLX subscales for the three agricultural tasks (baling, rotary tilling, and sowing).

The subjective cognitive workload, quantified by the NASA-TLX across six subscales, exhibited a highly heterogeneous distribution dependent on the specific agricultural task properties. The multidimensional assessment scores are visualized in **Figure 2**.

Statistical analysis revealed a significant main effect of task type on Mental Demand ( $F(2,\;22)=45.32,\;\;\;p<0.001,\;\;\;\eta_{p}^{2}=0.68$). *Post hoc* comparisons indicated that baling induced significantly higher mental demand ( 82.5±5.4) compared to rotary tilling ( 41.2±6.1,   p<0.001) and sowing ( 58.4±7.2,   p<0.01). A similar distinct pattern was observed in Temporal Demand ( F(2, 22)=38.15,   p<0.001, ) and Frustration ( F(2, 22)=29.84,   p<0.001, ), where baling consistently imposed the highest burden. This subjective profile aligns with the continuous mechanical monitoring and precise trajectory alignments required during baling operations, characterizing it as a state of active information overload.

Conversely, rotary tilling elicited a significantly higher Physical Demand compared to the other two tasks ( F(2, 22)=18.67,   p<0.01), while its Mental Demand and Temporal Demand were the lowest. The subjective responses suggest that rotary tilling constitutes a physically constrained but cognitively passive task, characterized by repetitive and monotonous operational features. Sowing consistently occupied the intermediate tier across most cognitive dimensions, representing a balanced workload state.

### 3.2 Bidirectional divergence and nonlinear evolution of visual channel responses

**Figure 3:**
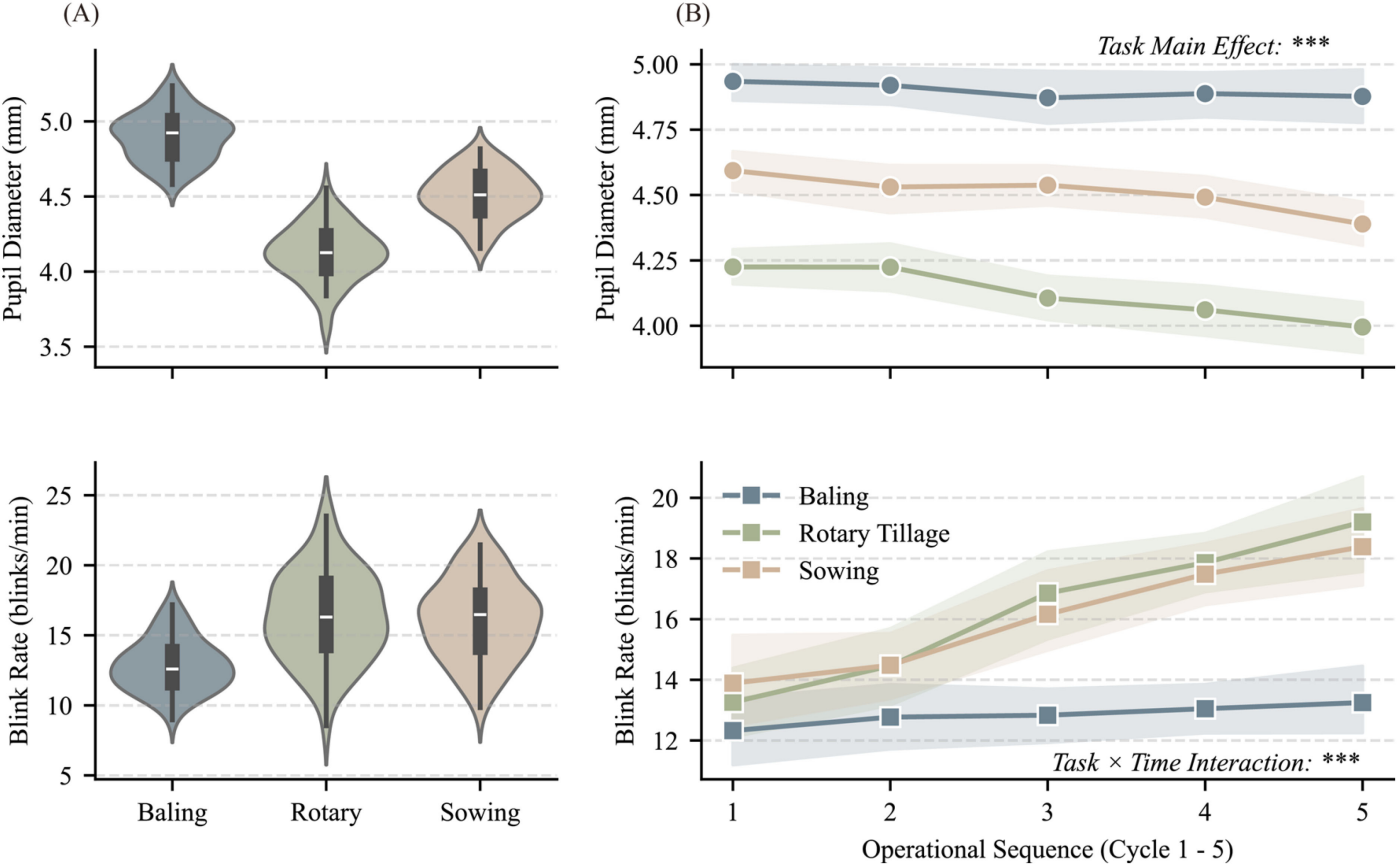
Bidirectional divergence and nonlinear temporal evolution of visual channel responses. **(A)** Probability density distributions of pupil diameter and blink rate across the three agricultural tasks (N = 12). Violin plots with internal boxplots depict inter-subject variance. **(B)** Longitudinal trajectories of visual metrics across the 5-hour operational cycles. Shaded bands represent the 95% confidence intervals (CI). *p* < 0.001***.

The objective visual channel responses exhibited a pronounced bidirectional divergence, associated with the specific perceptual demands of the agricultural tasks under the fixed cyclic protocol. To capture both the underlying data distribution and the temporal dynamics, the probability densities and the longitudinal evolution trajectories of pupil diameter and blink rate are presented in **Figure 3**.

Analysis of pupil diameter, an ocular indicator associated with cognitive arousal and mental workload, showed a significant main effect of Task ( $F(2,\;22)=18.54,\;\;p<0.001^{***},\;\;\;\eta_{p}^{2}=0.62$). As illustrated in the distributional analysis (**Figure 3A**), baling operations were associated with a highly concentrated state of maximal pupillary dilation (overall mean 4.85 ± 0.31 mm). The temporal trajectory (**Figure 3B**), top panel) demonstrates that this dilation remained sustained near the physiological ceiling across all five cycles, exhibiting minimal degradation. This sustained structural expansion may reflect a continuous state of active information processing and high vigilance, as operators were forced to relentlessly monitor complex rear-implement statuses and align field trajectories, preventing the visual system from habituating.

In stark contrast, the spontaneous blink rate—an ocular-behavioral indicator related to visual fatigue and task disengagement—displayed a diametrically opposed, nonlinear trajectory. A robust Task × Time interaction was observed for blink frequency ( $F(8,\;88)=14.32,\;\;p<0.001^{**},\;\;\;\eta_{p}^{2}=0.54$). During rotary tilling, the blink rate not only exhibited the highest overall variance (**Figure 3A**) but also manifested a profound exponential surge beginning at Cycle 3, ultimately peaking at 24.6 ± 3.2 blinks/min by Cycle 5 (**Figure 3B**), bottom panel). This hyper-frequent blinking, coupled with concurrent pupillary constriction (*p* < 0.01 relative to baling), suggests a fundamental shift in visual-cognitive strategy. Deprived of dynamic external stimuli during the highly monotonic rotary tilling process, the visual channel experienced passive perceptual withdrawal. The accelerated blink rate is consistent with passive perceptual withdrawal and may indicate the onset of severe passive drowsiness and visual disengagement. Sowing maintained an intermediate, linear trajectory across both metrics, reflecting a balanced, sustainable state of visual-motor coordination.

### 3.3 Antagonistic evolution of central and autonomic nervous system mechanisms

**Figure 4:**
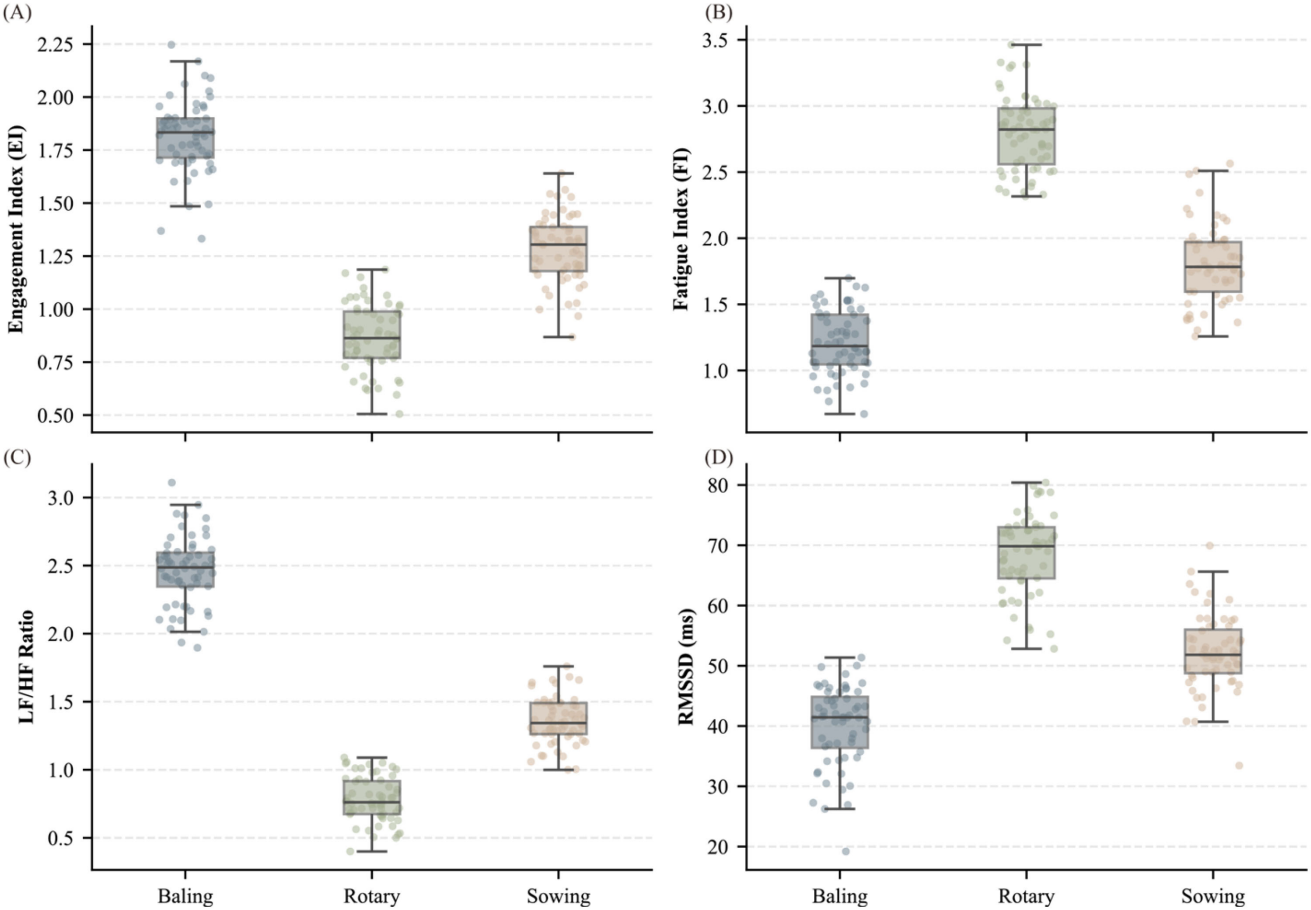
High-dimensional distribution of core “brain-heart” neurophysiological features across the three agricultural tasks (N = 12). Hybrid plots display boxplots (median and interquartile ranges) combined with individual data point scattering for **(A)** Engagement Index (EI), **(B)** Fatigue Index (FI), **(C)** LF/HF ratio, and **(D)** RMSSD.

**Figure 5:**
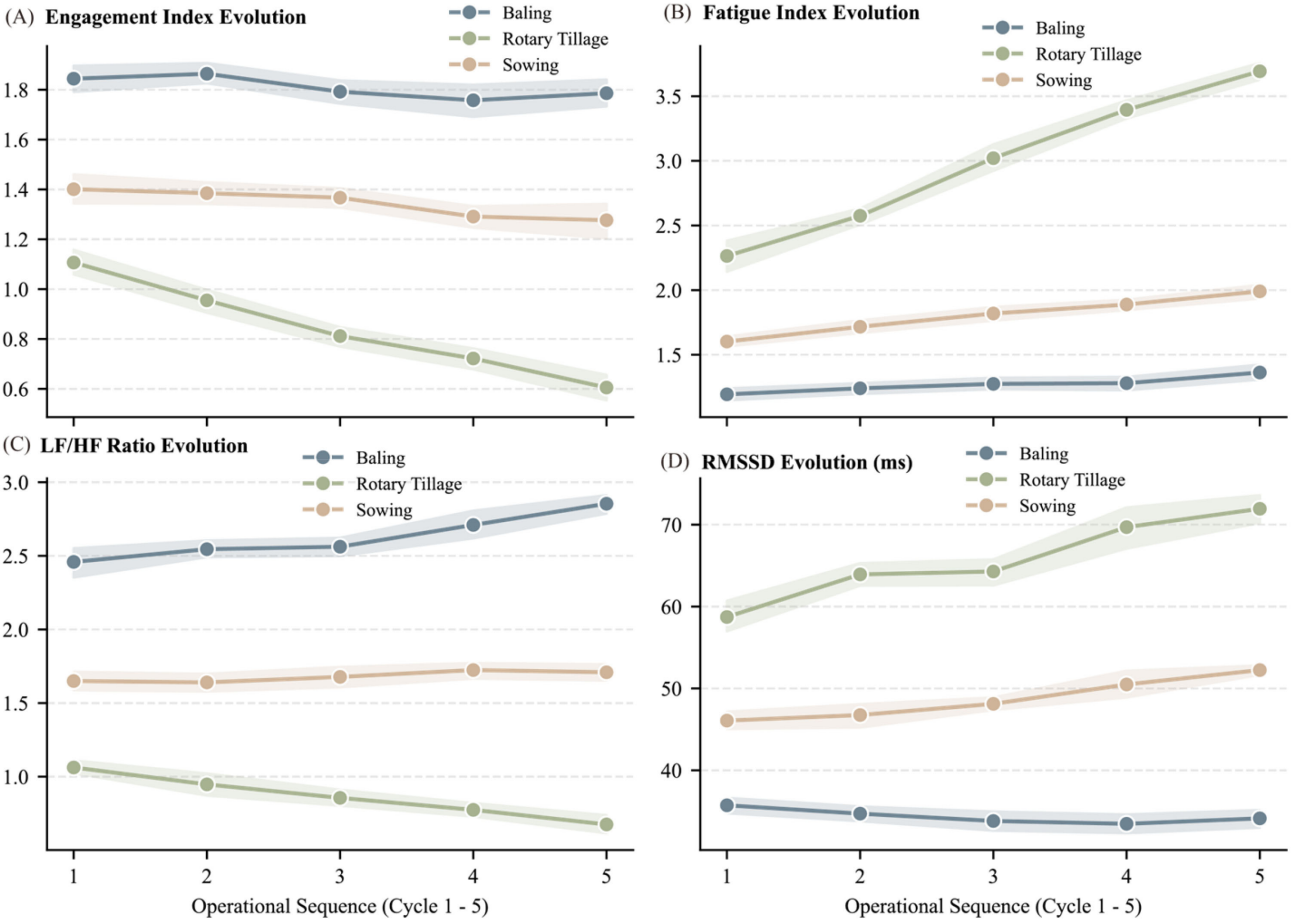
Temporal evolution of central and autonomic physiological metrics over the continuous 5-hour driving protocol: **(A)** Engagement Index (EI), **(B)** Fatigue Index (FI), **(C)** LF/HF ratio, and **(D)** RMSSD. Solid lines denote group means, and shaded regions represent the 95% confidence intervals (CI).

The synergistic analysis of central cortical oscillations and autonomic cardiac rhythms showed task-dependent differences in central and autonomic response patterns between different agricultural tasks. The distributions of the core “brain-heart” neurophysiological features and their longitudinal evolution trajectories are visually decoupled in **Figures 4**, **5**, respectively.

During baling operations, the human-machine interaction is characterized by continuous mechanical monitoring, complex implement alignment, and frequent anomaly interventions. This high-density perceptual demand was associated with concurrent increases in both the Engagement Index (EI) and the Low-Frequency to High-Frequency (LF/HF) ratio. Statistical modeling confirmed a robust main effect of Task on EI ( $F(2,\;22)=24.18,\;\;\;\;p<0.001,\;\;\;\eta_{p}^{2}=0.68$), and *LF/HF* ( F(2, 22)=19.45,    p<0.001). The concurrent elevation of these metrics supports the interpretation of an active-load-dominant fatigue pattern. Biologically, the increase in beta-band activity is consistent with sustained attentional engagement and active cognitive processing, while the elevated LF/HF ratio suggests increased sympathetic modulation. The temporal evolution (**Figures 5A, C**) demonstrates that central-autonomic activation pattern remained elevated across all five cycles, suggesting that fatigue during baling may be characterized more by sustained task engagement and physiological activation than by a classical vigilance-decrement pattern.

Conversely, the physically constrained yet cognitively monotonous nature of rotary tilling was associated with a different central-autonomic response pattern. Rotary tilling induced significantly higher levels of the Fatigue Index (FI) and the Root Mean Square of Successive Differences (RMSSD) compared to baling (*p* < 0.001). The robust Task × Time interaction for FI (​ $F(8,\;88)=16.72,\;\;\;\;p<0.001,\;\;\;\eta_{p}^{2}=0.60$) and RMSSD ( $F(8,\;88)=13.55,\;\;\;\;p<0.001^{***}$) suggests that the passive-fatigue-related response evolved progressively over repeated cycles. Deprived of variable external stimuli, the central nervous system showed an increase in theta and alpha spectral activity, marked by a surge in theta and alpha spectral power (**Figure 5B**). Simultaneously, the autonomic nervous system showed increased parasympathetic-related activity, reflected by the exponential expansion of the RMSSD time-domain variance (**Figure 5D**). This specific “high FI - high RMSSD” multimodal fingerprint is consistent with a passive-deprivation-dominant fatigue pattern characterized by reduced vigilance tendency and visual-task disengagement.

### 3.4 Spatiotemporal topography and spectral dynamics of cortical oscillations

The spatiotemporal distribution of cortical activation and its corresponding power spectral density (PSD) exhibited significant task-dependent divergences across different agricultural operational modes. To comprehensively map the central nervous system (CNS) responses, topographical matrices and regional PSD analyses were extracted to fingerprint the distinct fatigue pathways.

**Figure 6:**
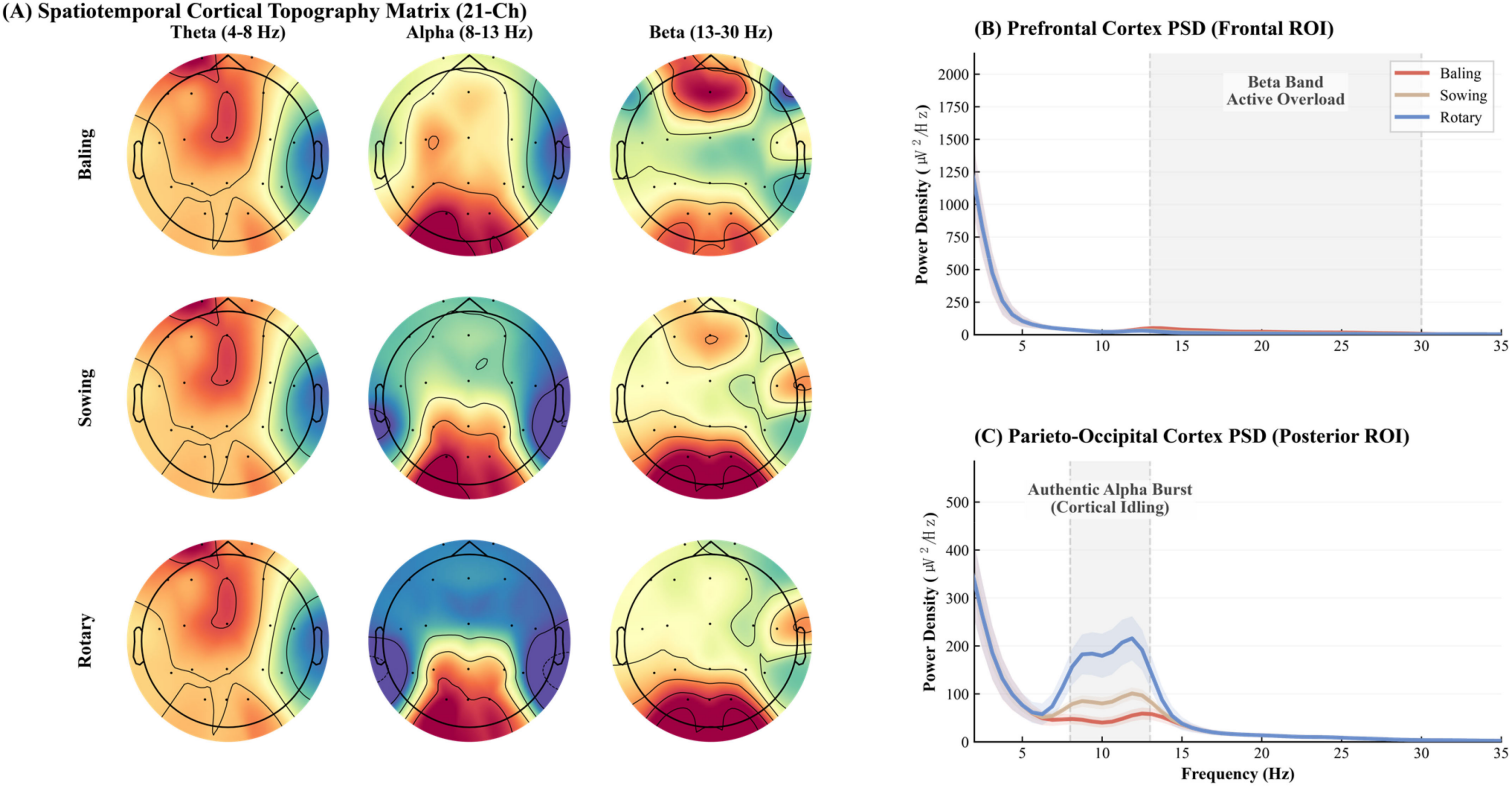
Spatiotemporal dynamics of cortical activation and regional power spectral density (PSD) across distinct agricultural tasks. **(A)** 21-channel EEG topographical matrix illustrating the spatial distribution of Theta (4–8 Hz), Alpha (8–13 Hz), and Beta (13–30 Hz) frequency bands during baling, sowing, and rotary tilling. **(B)** Grand average PSD curves over the prefrontal cortex (frontal ROI: Fp1, Fpz, Fp2, F3, Fz, F4), with the Beta band (13–30 Hz) highlighted. **(C)** Grand average PSD curves over the parieto-occipital cortex (posterior ROI: P3, Pz, P4, O1, Oz, O2), with the Alpha band (8–13 Hz) highlighted. In **(B, C)**, solid lines represent group means (N = 12) and translucent shaded regions denote the standard error of the mean (SEM). (* *p* < 0.05).

As illustrated in the topographical matrix (**Figure 6A**), the baling task elicited a pronounced synchronization of Beta-band power (13–30 Hz), primarily localized in the prefrontal and frontal cortices. The region-of-interest (ROI) PSD analysis over the frontal region (**Figure 6B**) further revealed a significant elevation in high-frequency spectral power during baling compared to other tasks. This highly localized anterior neural activation indicates continuous top-down attentional allocation, characterizing a state of active information overload induced by high-frequency target monitoring.

Conversely, the rotary tilling task was fundamentally characterized by substantial Alpha-band (8–13 Hz) hypersynchrony, which expanded massively across the parieto-occipital regions. The parieto-occipital PSD curve (**Figure 6C**) exhibited a prominent authentic alpha burst peaking around 10 Hz. This posterior alpha enhancement is consistent with cortical idling and reduced external-task engagement; however, it should not be interpreted as direct evidence of DMN intrusion in the absence of functional connectivity or neuroimaging measures. Together with the visual and autonomic indicators, this pattern suggests that the monotonous nature of continuous tilling was associated with a passive-deprivation-like state. Repeated measures ANOVA showed the main effect of task modality on the localized spectral power of both Alpha and Beta bands (all * *p* < 0.05). The transitional sowing task consistently elicited intermediate spectral values, mapping the progressive neural shift between active engagement and passive cortical idling.

### 3.5 Statistical analysis summary

To systematically evaluate the main and interaction effects of operational task modality and time-on-task (cycle) on multimodal physiological indicators and subjective workload, a two-way repeated measures analysis of variance (RM-ANOVA) was conducted. Task modality (Baling, Sowing, Rotary tilling) and time-on-task (Cycles 1–5) were entered as within-subject factors. Greenhouse-Geisser corrections were applied to degrees of freedom (df) when the assumption of sphericity was violated (Mauchly’s test, *p* < 0.05). The effect size was determined using partial eta squared ( $\eta_{p}^{2}$).

The comprehensive statistical results are summarized in **Table 3**. Task modality exhibited a highly significant main effect across all multimodal metrics (all *p* < 0.001), suggesting that different agricultural operations were associated with distinct neural, visual, and autonomic response patterns within the present cohort. Furthermore, the main effect of time-on-task was significant for fatigue-related indices (e.g., Blink Rate, RMSSD, and Fatigue Index), indicating time-related changes in fatigue-associated physiological indicators. Notably, significant Task × Time interaction effects were observed in the autonomic and visual channels, indicating that the trajectory of fatigue accumulation is potentially task-dependent, with rotary tilling accelerating parasympathetic dominance and visual fatigue more rapidly than other tasks.

**Table 3:** Two-way RM-ANOVA summary for multimodal physiological and subjective metrics.

Metrics/variables	Task (df=2, 22)		Time (df=4, 44)		Task × Time (df=8, 88)	
	*F*	*ηp* ^2^	*F*	*ηp* ^2^	*F*	*ηp* ^2^
Subjective						
NASA-TLX Score	45.32***	0.68	28.14***	0.52	12.05***	0.38
Eye-Tracking						
Pupil Diameter	18.54***	0.62	5.82*	0.22	3.45*	0.16
Blink Rate	32.15***	0.65	24.33***	0.58	14.32***	0.54
ECG/ANS						
LF/HF Ratio	19.45***	0.59	8.12**	0.28	5.21*	0.21
RMSSD	28.08***	0.64	16.45***	0.51	13.55***	0.49
EEG/CNS						
Engagement (EI)	24.18***	0.68	14.52**	0.41	4.88*	0.18
Fatigue Index (FI)	35.64***	0.69	28.22***	0.59	16.72***	0.60
Frontal Beta	22.77***	0.61	7.34**	0.26	3.12*	0.15
Occipital Alpha	38.54***	0.72	22.18***	0.55	11.45***	0.42

*p < 0.05, **p < 0.01, ***p < 0.001.

To further assess the robustness of the statistical findings under the small-sample design, additional sensitivity analyses were performed. The *post hoc* sensitivity power analysis indicated that the present within-subject design with 12 participants was mainly sensitive to large effects, with a minimum detectable effect size of *f* = 0.41, corresponding to $\eta_{p}^{2}$ = 0.14 at α = 0.05 and 1−β = 0.80. Bootstrap confidence intervals for representative task-related contrasts showed directionally consistent patterns, including higher pupil diameter during baling than rotary tilling, mean difference = 0.73, 95% CI [0.45, 1.01], and higher blink rate during rotary tilling than baling, mean difference = 4.7, 95% CI [2.1, 7.3]. Complementary linear mixed-effects models showed task-related patterns broadly consistent with the RM-ANOVA results. Subject-level consistency analysis further indicated that the expected task-related directions were observed in most participants, ranging from 10/12 to 11/12 across the primary physiological indicators. These robustness analyses provide sensitivity evidence for the within-cohort consistency of the main patterns, while the findings remain preliminary and require validation in larger and more diverse cohorts.

Because the three tasks were presented in a fixed cyclic order, additional sensitivity analyses were conducted to examine whether the main task-associated patterns were solely attributable to temporal order or fatigue accumulation. Cycle-wise consistency checks showed that the expected task-associated directions were preserved across 5/5 cycles for the active-overload indicators and across 5/5 cycles for the passive-deprivation indicators. A first-cycle-only sensitivity analysis further showed that the main directional patterns were already observable in Cycle 1 for 8/8 primary indicators, suggesting that the observed differences were not entirely explained by late-stage fatigue accumulation. Complementary mixed-effects models including temporal progression yielded fixed-effect directions broadly consistent with the main RM-ANOVA patterns. Nevertheless, because the task sequence was not randomized or counterbalanced, residual order and carryover effects cannot be fully excluded. Therefore, the results were interpreted as task-associated physiological patterns under the present fixed cyclic protocol rather than pure task effects.

### 3.6 Multimodal cross-channel coupling

To examine the associations among the central nervous system, autonomic responses, visual behaviors, and subjective perceived workload, a cross-channel Spearman rank correlation analysis was executed. By integrating the multidimensional metrics extracted from all experimental cycles, task-associated multimodal association patterns were observed within the present cohort.

**Figure 7:**
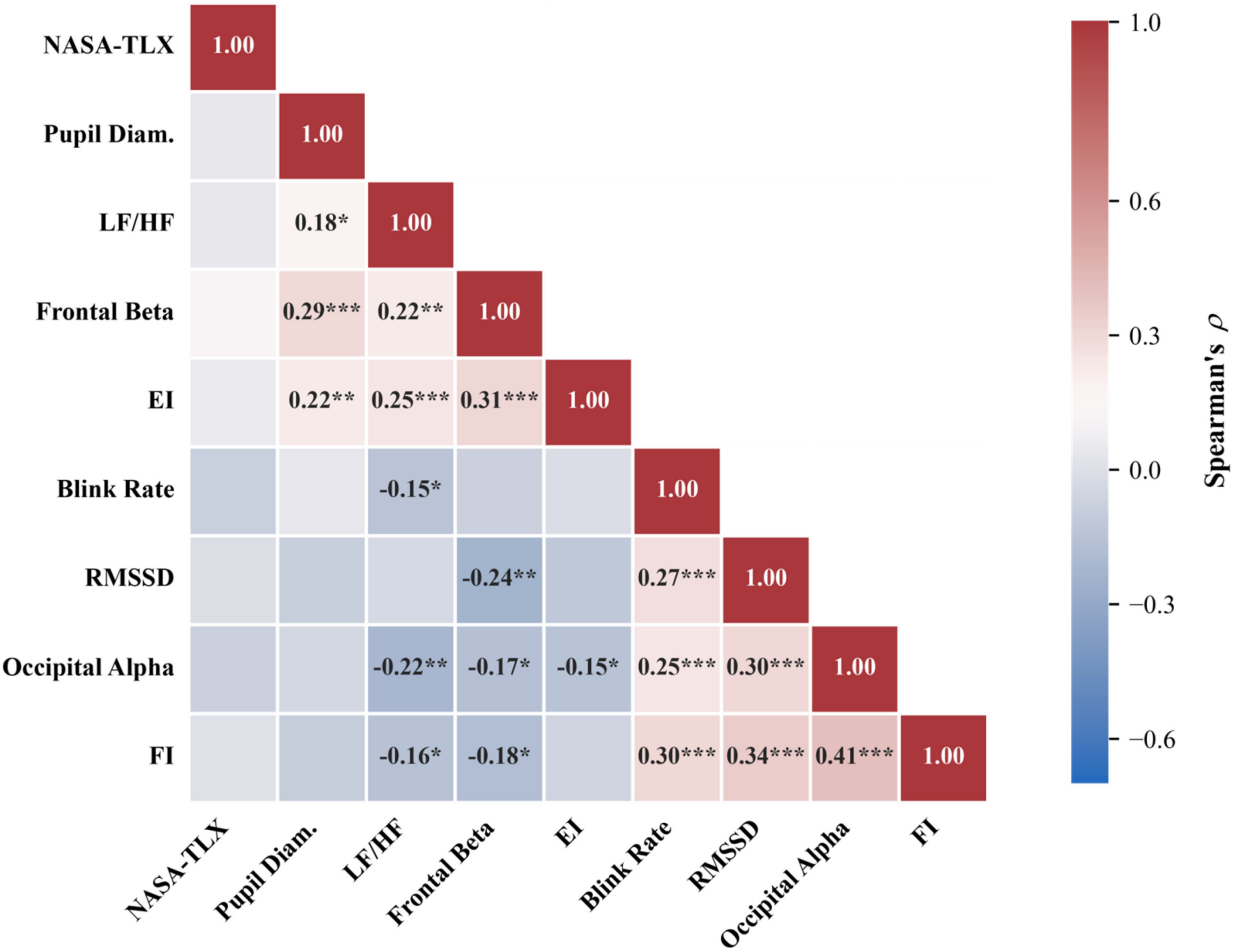
Multimodal cross-channel coupling analysis. The lower-triangle heatmap illustrates the Spearman rank-order correlation coefficients among the subjective NASA-TLX scores, visual (pupil diameter, blink rate), central (EI, FI, frontal Beta, parieto-occipital Alpha), and autonomic (LF/HF ratio, RMSSD) metrics. Asterisks indicate statistical significance after FDR correction: *p < 0.05, **p < 0.01, ***p < 0.001.

As depicted in the lower-triangle correlation heatmap (**Figure 7**), two distinct physiological clusters emerged, showing feature-grouping patterns broadly consistent with the proposed active-overload-like and passive-deprivation-like framework. The active overload indicators exhibited robust positive intra-cluster correlations: subjective NASA-TLX scores were significantly coupled with the Engagement Index (EI), frontal Beta power, pupil diameter, and the LF/HF ratio (correlation coefficients $r_{s}$ ranging from 0.38 to 0.62, all *p* < 0.01). This multi-system convergence suggests that higher subjective workload was accompanied by coordinated increases in attentional-engagement-related EEG activity, pupil diameter, and sympathetic-related autonomic activity.

Conversely, metrics characterizing passive deprivation formed a consistent secondary cluster. The Fatigue Index (FI) demonstrated significant positive correlations with parieto-occipital Alpha power, RMSSD, and blink rate ( $r_{s}\in [0.42,\;\;0.65],\;\;p<0.01$), indicating that EEG spectral slowing-related indicators, parasympathetic-related activity, and visual disengagement-related behavior tended to co-vary under monotonous task conditions. Furthermore, moderate but significant negative cross-correlations were observed between the two extreme clusters (e.g., EI vs. FI, $r_{s}=-0.55$; LF/HF vs. RMSSD, $r_{s}=-0.48$), suggesting an inverse association between active-load-related and passive-deprivation-related physiological signatures. The robust alignment across the “eye-brain-heart” channels support the potential value of multimodal joint assessment for reducing the ambiguity of single-channel fatigue interpretation in agricultural ergonomic evaluation.

**Figure 8:**
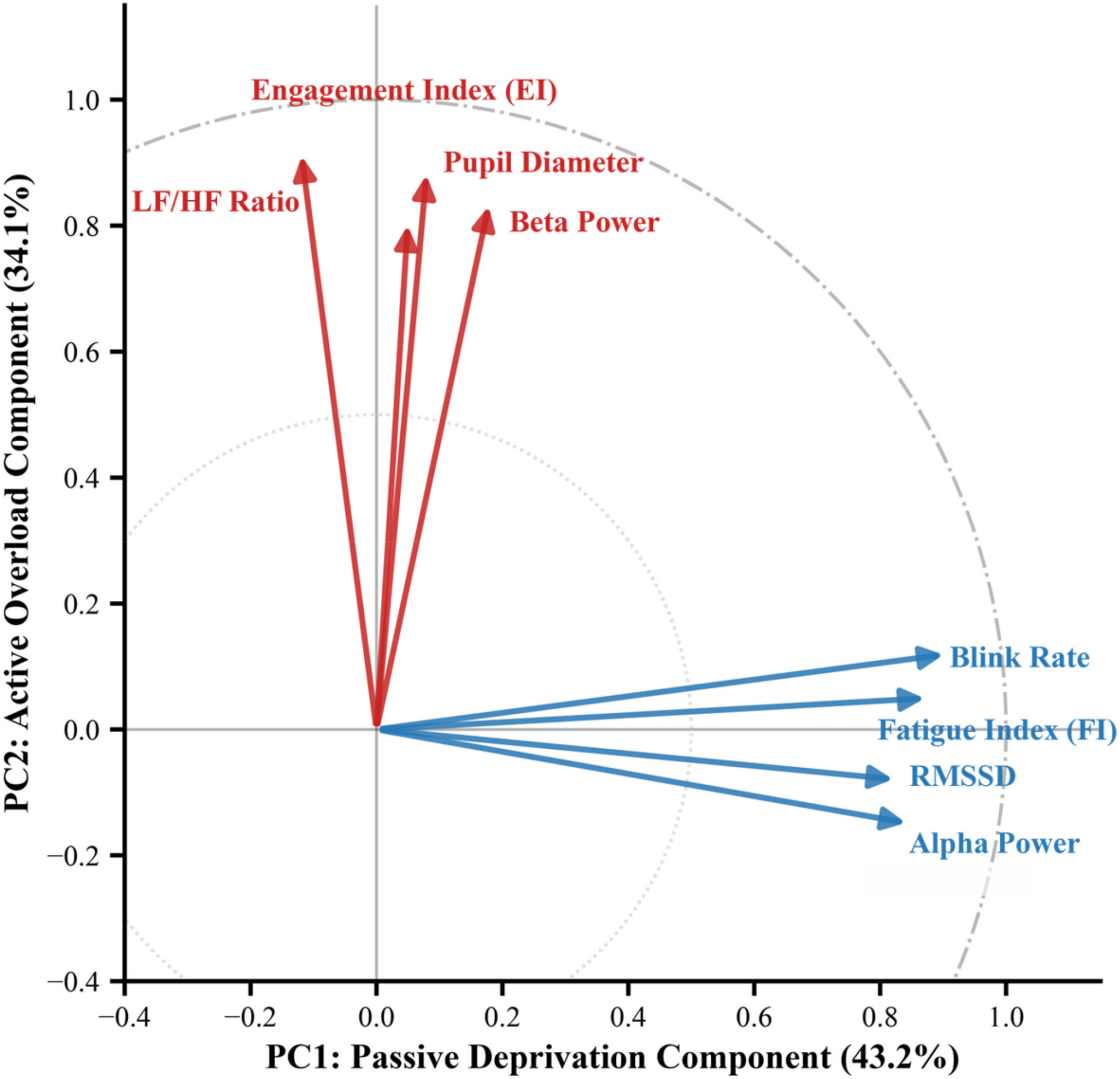
Principal Component Analysis (PCA) loading plot of the multimodal physiological features, cumulatively explaining 77.3% of the total variance. Variables are visually grouped along two exploratory components: the passive-deprivation-related indicators (RMSSD, Alpha power, FI, and blink rate) and the active-overload-related indicators (LF/HF, Beta power, EI, and pupil diameter). PCA was interpreted as exploratory dimensionality reduction rather than confirmatory validation of latent mechanisms.

To further explore these cross-channel correlations dimensionally, the PCA extracted two primary principal components (PCs) with eigenvalues greater than 1.0, which cumulatively explained 77.3% of the total dataset variance. As vividly illustrated in the loading plot (**Figure 8**), a clear feature grouping pattern was observed. PC1, termed the ‘Passive Deprivation Component’, accounted for 43.2% of the variance and exhibited heavy positive loadings from the Fatigue Index (FI), RMSSD, spontaneous blink rate, and parieto-occipital Alpha power. PC2, conceptualized as the ‘Active Overload Component’ (34.1% of variance), was mainly characterized by high positive loadings from the Engagement Index (EI), LF/HF ratio, frontal Beta power, and pupil diameter. The distinct clustering of these high-dimensional features into two feature groups was interpreted as an exploratory dimensionality-reduction result, suggesting that the multimodal features tended to organize along two interpretable axes consistent with the proposed active-overload and passive-deprivation framework.

Given the limited sample size, PCA was interpreted as exploratory dimensionality reduction rather than confirmatory validation. Leave-one-subject-out PCA showed that the two-component structure was generally preserved, with cumulative explained variance ranging from 74.2% to 79.5% across the 12 iterations. The main loading directions of the active-overload and passive-deprivation indicators were preserved in 12/12 iterations, suggesting that the exploratory PCA pattern was not dominated by a single participant.

## 4 Discussion

### 4.1 Task properties are associated with divergent fatigue-related physiological patterns

The primary objective of this study was to investigate the multidimensional cognitive workload and fatigue evolution during sustained agricultural operations. Contrary to the traditional ergonomic view that models driving fatigue as a monolithic, time-dependent vigilance decrement, the multimodal physiological evidence presented herein provides preliminary within-cohort evidence consistent with task-dependent divergence in fatigue-related physiological patterns. The intrinsic properties of the agricultural task may influence whether the operator’s central and autonomic nervous systems transition toward a state of active information overload or passive monotonous deprivation. However, because the tasks were presented in a fixed cyclic order, these task-associated patterns should be interpreted within the present experimental protocol, and residual order, carryover, and time-lapse effects cannot be fully excluded.

During the baling operation, the necessity for continuous trajectory alignment and high-frequency rear-implement monitoring imposed severe mental and temporal demands. This high-density perceptual requirement elicited a synchronized arousal state, characterized by sustained prefrontal Beta synchronization and an elevated LF/HF ratio. The persistent pupillary dilation observed throughout the baling cycles is consistent with sustained cognitive arousal, and may be compatible with noradrenergic arousal processes reported in previous studies. Previous studies have linked task-evoked pupil dilation to LC-related noradrenergic activity during mental effort; however, the present study did not directly measure LC-NE activity, and pupil dilation was therefore interpreted only as an indirect ocular proxy of cognitive arousal (Elman et al., 2017; Granholm et al., 2017). Unlike typical highway driving—which primarily induces passive underload—baling forces the operator into a relentless top-down attentional allocation loop. Consequently, while this sympathetic dominance temporarily inhibits the onset of classical physical drowsiness, it accelerates acute cognitive stress and the depletion of executive attentional resources. To theoretically contextualize this sustained hyperarousal over the prolonged 5-hour protocol, these findings can be cautiously interpreted through the lens of the Compensatory Control Theory and the Cognitive Resource Depletion model. The absence of a classical vigilance decrement—typically anticipated with extended time-on-task—during baling does not imply an absence of fatigue. Rather, the rigidly elevated arousal-related physiological pattern, coupled with prefrontal Beta synchronization, indicates a high-cost neurophysiological compensatory mechanism. To meet the stringent spatial tracking and implement-alignment demands, operators must continuously exert explicit top-down cognitive effort, effectively forcing the autonomic and central nervous systems into an “active override” state. While this intense sympathetic drive successfully masks overt physical sleepiness, it heavily taxes the brain’s finite metabolic and executive capacities. This mechanistic explanation may help link the objective “brain-heart” hyperarousal with the profoundly elevated subjective Frustration and Mental Demand scores observed in the NASA-TLX, suggesting that time-on-task during complex agricultural operations manifests as a high-cost latent active exhaustion, fundamentally distinct from passive drowsiness.

In stark contrast, rotary tilling represents a paradigm of passive task deprivation. The highly monotonous operational environment, physically constrained yet devoid of variable external stimuli, triggered a distinct fatigue-related physiological pattern. The prominent parieto-occipital Alpha burst—a recognized hallmark of cortical idling—symmetrically coincided with an exponential surge in spontaneous blink rate and vagal-dominated RMSSD elevation. This cross-channel convergence is consistent with passive disengagement and may reflect task-unrelated processing tendencies that have been associated with DMN activity in prior studies. However, posterior alpha enhancement alone cannot identify DMN intrusion, and direct confirmation would require functional connectivity or neuroimaging evidence. When external task demands fall below the optimal threshold of the human cognitive envelope, the central nervous system reflexively shifts toward internal, task-unrelated processing, leading to an insidious decrement in vigilance. Boredom and disengagement from monotonous tasks are marked by DMN activation (Danckert and Merrifield, 2018), and growing mental fatigue produces a shift away from executive networks to the default mode, accompanied by a shift in alpha frequency bands (Trejo et al., 2015). The exponential trajectory of the visual and EEG fatigue indices during rotary tilling suggests that prolonged task underload may pose a potential threat to operational safety.

The transitional physiological state observed during the sowing task effectively maps the continuum between these two physiological extremes. Collectively, these findings suggest that, within the present cohort, time-on-task alone is an insufficient predictor of agricultural operational fatigue. Accurate fatigue modeling may benefit from incorporating the specific cognitive properties of the executed task.

### 4.2 Potential value of the “eye-brain-heart” multimodal fingerprint

Traditional ergonomic evaluations of agricultural operators have predominantly relied on *post-hoc* subjective questionnaires or unidimensional physiological metrics. While subjective tools like the NASA-TLX provide valuable psychological insights, they are inherently limited by recall bias, subjective scaling inconsistencies, and an inability to capture real-time dynamic shifts. Similarly, relying on a single physiological channel often yields ambiguous interpretations. For instance, heart rate variability alone cannot distinguish between physical exertion and mental stress, while EEG—despite its central accuracy in detecting shifts in cognitive processes and alpha/theta wave alterations (Awais et al., 2017)—is susceptible to motion artifacts in field environments.

The findings of this study suggest the potential value of integrating the “Eye-Brain-Heart” multimodal physiological fingerprint. Recent systematic reviews in driver fatigue detection emphasize the necessity of multimodal integration to bridge the gap between experimental models and real-world monitoring systems (Awais et al., 2017; Ajayi et al., 2025). As evidenced by the cross-channel coupling analysis (**Figure 7**), these three physiological subsystems do not operate in isolation but function as a coordinated neuroergonomic response pattern within the present dataset.

The visual channel (Eye) serves as the primary interface for environmental interaction, providing immediate biomarkers of perceptual engagement (pupillary dilation) and visual disengagement, such as increased blink frequency captured by eye-tracking (Ajayi et al., 2025). The central nervous system (Brain), captured via EEG, provides direct electrophysiological evidence of cortical oscillatory activity, allowing task-related changes in beta and alpha/theta spectral patterns to be examined as proxy indicators of attentional engagement and fatigue-related cortical slowing. Concurrently, the autonomic nervous system (Heart) provides the foundational metabolic and autonomic rhythm, revealing the sympathetic or parasympathetic tone that sustains the operator’s physiological state.

By fusing these modalities, the proposed “Eye-Brain-Heart” fingerprint may reduce some limitations of isolated metrics. The observed cross-channel correlations—such as the alignment of frontal Beta power (Brain), elevated LF/HF ratio (Heart), and continuous pupil dilation (Eye) during baling—create a convergent physiological pattern. This multidimensional cross-channel convergence may help reduce the physiological ambiguity present in single-channel assessments. Ultimately, this multimodal framework affords a comprehensive, mechanistically interpretable assessment approach, which may be useful for capturing the nuanced, dual-pathway nature of operational fatigue in complex agricultural human-machine systems.

### 4.3 Implications for future adaptive agricultural HMI

The overarching objective of establishing this multimodal physiological fingerprint is to facilitate a paradigm shift from passive operator monitoring to closed-loop, adaptive human-machine interaction (HMI). Current agricultural tractor cabins predominantly employ static, monolithic interfaces that are ill-equipped to accommodate the dynamically fluctuating cognitive states of operators. Based on the task-associated physiological patterns observed in this exploratory study, future intelligent HMI systems may consider context-aware intervention strategies, although direct experimental validation is still required. Cognitive-adaptive interfaces can be engineered to dynamically recalibrate system behavior in response to the operator’s real-time physiological state, thereby balancing workload and sustaining optimal decision-making capacity. Therefore, the interface-related proposals in this section should be regarded as candidate design implications derived from the observed physiological patterns, rather than experimentally validated intervention strategies. Before practical deployment, these candidates should be examined through intermediate steps, including offline physiological-state mapping, prototype implementation, closed-loop simulator testing, and field validation. Cognitive-adaptive interfaces are designed to adjust their behavior dynamically to accommodate the changing needs, context, and abilities of the user, thereby balancing workload and enhancing decision-making (Keinrath et al., 2010; Huttner et al., 2024; Meftah et al., 2026).

For active overload scenarios, such as baling operations—where the operator is subjected to sustained high-frequency perceptual demands and sympathetic hyperarousal—the adaptive HMI strategy could prioritize “sensory attenuation and task offloading.” When the multimodal monitoring system detects sustained prefrontal Beta synchronization coupled with continuously dilated pupils and an elevated LF/HF ratio, the intelligent cabin could initiate a “decluttering” mode. This involves suppressing non-critical information on the Head-Up Display (HUD) to mitigate cognitive overload and minimize visual search time (Huttner et al., 2024). Furthermore, the system should dynamically elevate the level of autonomous intervention—such as activating predictive implement alignment or automated PTO (Power Take-Off) speed control—to effectively buffer the operator’s mental demand and prevent the exhaustion of executive resources.

Conversely, during passive deprivation states typical of rotary tilling—where cortical idling (Alpha bursts) and parasympathetic dominance (high RMSSD) prevail—the HMI could evaluate “sensory augmentation and vigilance restoration.” To counter passive disengagement and reduced external-task vigilance, the system may help interrupt the monotonous environmental feedback loop. Upon detecting an exponential surge in blink rate alongside parieto-occipital Alpha hypersynchrony, the HMI should introduce targeted, multisensory stimuli. This can be achieved through variable haptic seat vibrations, directional auditory alerts, or even the introduction of cognitively stimulating secondary tasks. Additionally, the system could enforce intermittent manual override intervals, encouraging the operator to actively re-engage with the control loop, thereby artificially restoring an optimal level of cognitive arousal.

### 4.4 Limitations and future prospects

While this study provides a comprehensive neuro-ergonomic framework for agricultural fatigue, several limitations must be acknowledged. First, the experiment was conducted in a highly controlled simulator environment. Although this design effectively eliminated environmental confounders (e.g., luminance variations) to isolate the cognitive effects of task properties, it inherently lacked the complex physical stressors present in actual field operations, such as whole-body vibration, extreme thermal conditions, and unpredictable terrain dynamics. These physical stressors may interact with cognitive fatigue in non-linear ways.

Second, the participant cohort was restricted to 12 experienced male operators. Although the within-subject design and within-subject normalization reduced inter-individual baseline variability, they did not eliminate the limitations associated with the small sample size, reduced statistical power, and restricted generalizability. This demographic homogeneity limits the generalizability of the findings across varying age groups, genders, and experience levels. In particular, because no *a priori* power analysis was conducted before participant recruitment, the present results should be interpreted as preliminary within-cohort evidence rather than definitive validation of a generalizable fatigue model. The *post hoc* sensitivity power analysis indicated that the current design was mainly sensitive to large within-subject effects; therefore, smaller effects and interaction effects may have been underpowered. In addition, the use of RM-ANOVA interaction terms and PCA in a small cohort may increase the risk of effect-size overestimation and component instability, although supplementary bootstrap confidence intervals, mixed-effects models, subject-level consistency analysis, and leave-one-subject-out PCA were added to evaluate the robustness of the main patterns.

Third, the three agricultural tasks were presented in a fixed cyclic order rather than in a randomized or counterbalanced sequence. Although this design helped maintain a consistent experimental protocol, it may introduce potential confounding between task-associated physiological differences and temporal order, carryover effects, or time-lapse-related fatigue accumulation. Additional sensitivity analyses were conducted to examine whether the main directional patterns were preserved across cycles and were not solely attributable to late-stage fatigue accumulation; however, these analyses cannot fully separate task-specific effects from order-related influences. Therefore, the observed physiological differences should be interpreted as task-associated patterns under the present fixed cyclic protocol rather than as definitive evidence of order-independent task effects.

Fourth, the EEG-derived Engagement Index and Fatigue Index were operational spectral proxy indices based on theta, alpha, and beta band power, rather than direct measurements of latent neurocognitive constructs. Although these indices provide a theory-driven representation of task engagement and fatigue-related cortical slowing, they cannot by themselves establish the underlying neural pathways. Therefore, their mechanistic interpretation should be considered in conjunction with ocular and autonomic evidence, and future studies incorporating functional connectivity analyses or neuroimaging measures would further strengthen construct validation. More specifically, pupillary dilation and posterior alpha enhancement were treated as indirect physiological correlates of cognitive arousal and reduced external-task engagement, respectively; they cannot directly establish LC-NE activation or DMN intrusion without direct functional connectivity or neuroimaging evidence.

Future research must bridge the gap between laboratory simulations and real-world agricultural environments. Subsequent studies will focus on deploying wearable “Eye-Brain-Heart” sensor networks in actual field operations to test whether the task-associated physiological patterns observed in this exploratory study can be replicated under ecological conditions. Furthermore, translating the theoretical adaptive HMI strategies proposed in this study into functional software prototypes, closed-loop simulator tests, and field trials, and empirically evaluating their efficacy in mitigating operator fatigue remains a critical next step for advancing intelligent agricultural machinery. Larger, gender-diverse, and more heterogeneous cohorts should be recruited in future studies to evaluate the generalizability of the proposed multimodal fatigue signatures across broader agricultural operator populations. In addition, future experiments should use randomized or counterbalanced task sequences to more rigorously distinguish task-specific effects from temporal order and carryover effects.

## 5 Conclusion

This study explored the multidimensional cognitive workload and fatigue evolution in agricultural operations through a highly integrated “Eye-Brain-Heart” physiological framework. The comprehensive analysis provides preliminary within-cohort evidence that task properties may be associated with distinct fatigue-related physiological patterns. The main findings can be summarized as three core conclusions:

Task-associated fatigue-related physiological patterns: Agricultural driving fatigue is unlikely to be fully explained by a uniform time-dependent vigilance decrement alone. Complex, high-demand tasks (e.g., baling) induce a state of active information overload, characterized by sustained pupillary dilation, and sympathetic prefrontal Beta synchronization. Conversely, monotonous tasks, such as rotary tilling, were associated with passive-deprivation-related signatures, including increased blink rate, elevated RMSSD, and parieto-occipital Alpha enhancement consistent with task disengagement.

Potential value of multimodal joint assessment: The cross-channel coupling analysis suggests that the “Eye-Brain-Heart” physiological fingerprint may help reduce the diagnostic ambiguity inherent in single-modality evaluations. The observed cross-channel associations, such as FI, Alpha power, and RMSSD, indicate that perceptual, central, and autonomic nervous systems respond to operational demands as an indivisible neuro-ergonomic network.

Exploratory basis for future adaptive HMI: The observed active-overload- and passive-deprivation-related signatures provide an exploratory physiological basis for next-generation intelligent tractor cabins. By mapping real-time physiological fingerprints to specific cognitive states, future human-machine interfaces may be designed to dynamically adjust between sensory attenuation (task offloading) and sensory augmentation (vigilance restoration), thereby potentially improving both agricultural operational safety and efficiency. However, given the small male-only cohort and the fixed cyclic task sequence, these findings should be regarded as preliminary task-associated physiological signatures under the present protocol. Future studies with larger, gender-diverse, and more heterogeneous cohorts, as well as randomized or counterbalanced task orders, are required to evaluate the generalizability and order-independence of the proposed multimodal fatigue signatures.

## Data Availability

The raw data supporting the conclusions of this article will be made available by the authors, without undue reservation.
